# *l*-Tetrahydropalmatine, an Active Component of *Corydalis yanhusuo W.T. Wang*, Protects against Myocardial Ischaemia-Reperfusion Injury in Rats

**DOI:** 10.1371/journal.pone.0038627

**Published:** 2012-06-08

**Authors:** Yi Han, Wen Zhang, Yan Tang, Wenli Bai, Fan Yang, Liping Xie, Xiaozhen Li, Suming Zhou, Shiyang Pan, Qi Chen, Albert Ferro, Yong Ji

**Affiliations:** 1 Department of Geriatrics, the First Affiliated Hospital of Nanjing Medical University, Nanjing, China; 2 State Key Laboratory of Reproductive Medicine, Key Laboratory of Cardiovascular Disease and Molecular Intervention, Nanjing Medical University, Nanjing, China; 3 Department of Laboratory Medicine, The First Affiliated Hospital of Nanjing Medical University, Nanjing, China; 4 Department of Clinical Pharmacology, Cardiovascular Division, School of Medicine, King's College London, London, United Kingdom; University of Padova, Italy

## Abstract

*l*-Tetrahydropalmatine (*l*-THP) is an active ingredients of Corydalis *yanhusuo W.T. Wang*, which protects against acute global cerebral ischaemia-reperfusion injury. In this study, we show that *l*-THP is cardioprotective in myocardial ischaemia-reperfusion injury and examined the mechanism. Rats were treated with *l*-THP (0, 10, 20, 40 mg/kg b.w.) for 20 min before occlusion of the left anterior descending coronary artery and subjected to myocardial ischaemia-reperfusion (30 min/6 h). Compared with vehicle-treated animals, the infarct area/risk area (IA/RA) of *l*-THP (20, 40 mg/kg b.w.) treated rats was reduced, whilst *l*-THP (10 mg/kg b.w.) had no significant effect. Cardiac function was improved in *l*-THP-treated rats whilst plasma creatine kinase activity declined. Following treatment with *l*-THP (20 mg/kg b.w.), subunit of phosphatidylinositol 3-kinase p85, serine^473^ phosphorylation of Akt and serine^1177^ phosphorylation of endothelial NO synthase (eNOS) increased in myocardium, whilst expression of inducible NO synthase (iNOS) decreased. However, the expression of HIF-1α and VEGF were increased in I_30 min_R_6 h_, but decreased to normal level in I_30 min_R_24 h_, while treatment with *l*-THP (20 mg/kg b.w.) enhanced the levels of these two genes in I_30 min_R_24 h_. Production of NO in myocardium and plasma, activity of myeloperoxidase (MPO) in plasma and the expression of tumour necrosis factor-α (TNF-α) in myocardium were decreased by *l-*THP. TUNEL assay revealed that *l*-THP (20 mg/kg b.w.) reduced apoptosis in myocardium. Thus, we show that *l*-THP activates the PI3K/Akt/eNOS/NO pathway and increases expression of HIF-1α and VEGF, whilst depressing iNOS-derived NO production in myocardium. This effect may decrease the accumulation of inflammatory factors, including TNF-α and MPO, and lessen the extent of apoptosis, therefore contributing to the cardioprotective effects of *l*-THP in myocardial ischaemia-reperfusion injury.

## Introduction

Ischaemia/reperfusion (I/R) injury is an important complication of acute arterial occlusion and subsequent recanalisation; for example, following acute myocardial infarction, coronary artery recanalisation by thrombolytic therapy or percutaneous coronary intervention is used therapeutically in an attempt to minimize the infarct area. However the reoxygenation of the ischaemic heart leads to an area of myocardial loss of function [1], [2]. Many signaling molecules have been postulated to contribute to ischaemia-reperfusion injury including both reactive oxygen species and nitric oxide (NO).

NO, acting as a signaling molecule, plays a major regulatory role in several aspects of cellular function; for example, it causes vasodilatation, inhibits platelet function and leukocyte-endothelial interaction and modulates neurotransmission [3], [4], [5]. NO can also exert a potent anti-inflammatory effect, by suppressing adhesion molecule expression and cytokine release [6], [7]. Hypoxia-inducible factor-1 (HIF-1), is a transcription factor expressed in response to a decrease in the partial pressure of cellular oxygen. It is a heterodimer composed of α and β subunits. HIF-1α is stable in physiological condition and exquisitely sensitive to the onset of cellular hypoxic conditions, while HIF-1β is constitutive and not sensitive to hypoxia [8], [9], [10]. HIF-dependent genes, such as vascular endothelial growth factor (VEGF), are important in I/R injury via regulating collateral vessel development [9], [11].

**Figure 1:**
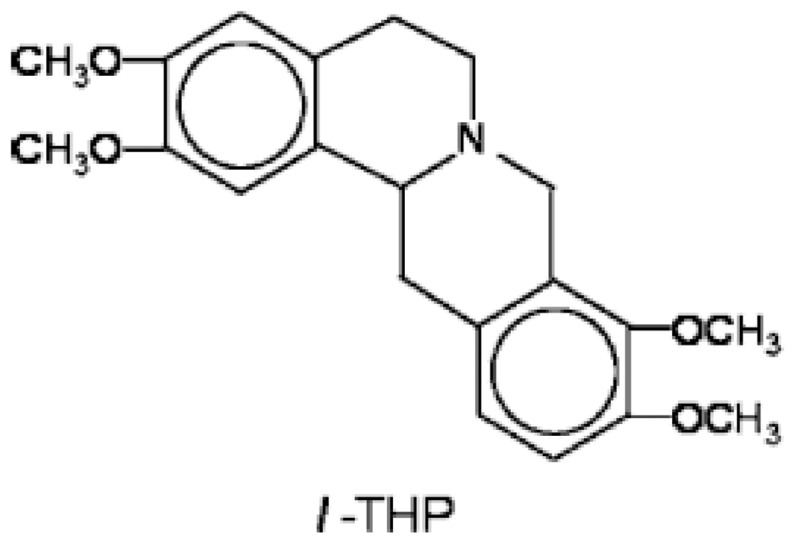
The chemical structure of *l*-THP.

Tetrahydroprotoberberines (THPBs) are a series of alkaloids, isolated from a Chinese analgesic medicine, called *Corydalis yanhusuo W. T. Wang*. *l*-Tetrahydropalmatine (*l*-THP), one of its main active ingredients, has been demonstrated to have potent analgesic effects and has been in use in Chinese clinical practice for this purpose for many years [12], [13]. Its chemical structure is shown in Figure 1. Studies have shown that giving *l*-THP, 2 or 30 min before ischaemia, can protect against acute global cerebral ischaemia-reperfusion injury in rats [14], [15]. *Corydalis yanhusuo W. T. Wang* has also been used in China for the treatment of a variety of cardiovascular diseases, and *l*-THP is once again believed to be the main active principle. More recent studies have suggested that another important mechanism of *l*-THP protection against global cerebral ischaemia-reperfusion injury is through reducing apoptosis by modulating the expression of heat shock protein 70, bcl-2 and bax [16], [17]. Additionally, ethanolic extracts of *Corydalis yanhusuo W. T. Wang* administered orally have been reported to protect against heart failure following induction of myocardial infarction in rats [18].

We hypothesized that *l*-THP protects the myocardium from ischaemia-reperfusion injury following acute coronary artery occlusion. The aim of the present study was therefore to investigate this possibility and to delineate the possible mechanisms by which *l*-THP is cardioprotective in this context.

## Methods

### Experimental animals and Ethics Statement

Male Sprague-Dawley rats (250–300 g) were purchased from the Laboratory Animal Resources of Nanjing Medical University (NJMU). The animals were fed a laboratory diet with water and food and kept under constant environmental conditions, with 12-hour light/dark cycles. Animal Resources at the NJMU were in accordance with the guidelines for the Principles of Laboratory Animal Care and the Guide for the Care and Use of Laboratory Animals. All aspects of animal care and all experimental protocols were approved by the NJMU Committee on Animal Care.

### Experimental model of myocardial ischaemia-reperfusion injury

Rats were anaesthetized with sodium pentobarbital (40 mg/kg intraperitoneally), given atropine (0.1 mg/kg subcutaneous injection) to reduce airway secretions, and artificially ventilated. Intraoperative monitoring of adequate anaesthesia is done by toe pinch. Myocardial ischaemia was induced by making a slipknot (6/0 silk) around the left anterior descending coronary artery (LAD), through a left thoracic incision. After 30 min ischaemia, the slipknot was loosened and the myocardium was reperfused for 6 h (I_30 min_R_6 h_) or 24 h (I_30 min_R_24 h_). 20 min prior to LAD ligation, rats were treated with either vehicle (same volume of Tris-HCl, pH 3.5) or *l*-THP (10, 20 or 40 mg/kg b.w.) by gavage, which ensures that *l*-THP could reach effective plasma concentration [15], [19], [20]. Sham-treated rats were given the same surgical operation without the slipknot and ligation-unlocked.

### Determination of myocardial infarct size

Myocardial infarct size was determined by Evans blue/triphenyltetrazolium chloride (TTC) staining as described previously [21], [22]. Briefly, for heart extraction, deep anaesthesia is achieved by overdose of sodium pentobarbital (60 mg/kg intraperitoneally) given at 0.2 ml/100 g b.w., the hearts were removed and perfused with saline using a Langendorff system, to wash blood from the coronary vasculature, followed by staining with 1.5% w/v Evans blue to determine the area at risk. The heart was then sliced horizontally into five slices, which were incubated in 1.2% TTC prepared with Tris Buffered Saline (TBS, pH 7.8) for 15 min at 37°C. Viable non-ischemic myocardium stains blue with Evans blue; ischemic but viable myocardium stains red with TTC; whilst necrotic myocardium does not stain with either and appears pale white. The infarct area (white) and the area at risk (red and white) from each section were determined using an AlphaEaseFC image analyzer (Alpha Innotech Corporation. CA, USA). Ratios of risk area to total left ventricle area (RA/LV) and infarct area to risk area (IA/RA) were calculated and expressed as percentages.

### Echocardiography

Rats were anaesthetized by inhalation with mild isoflurane (0.5–1.5%, Isoflurane Vaporizer, Matrx VIP3000, USA) and anchored to a positionable platform in a supine position. Cardiac function was evaluated by echocardiography using GE Vivid 7 equipped with a 14-MHz phase array linear transducer S12, allowing a 150 maximal sweep rate (General Electric Company, Connecticut, USA). All measurements were made by one observer who was blinded with respect to the identity of the treatments administered and were averaged over five consecutive cardiac cycles.

### Determination of creatine kinase activity

Plasma creatine kinase (CK) activity was determined spectrophotometrically at 340 nm by a commercial kit (Nanjing Jiancheng Bio-engineering Institute, China). In brief, CK catalyzes the phosphorylation of ADP, in the presence of creatine phosphate, to form ATP and creatine. The catalytic activity is determined from the rate of NADPH formation, measured by absorbance at 340 nm, by determining the hexokinase and glucose-6-phosphate dehydrogenase coupled reactions.

### Determination of myocardial and plasma myeloperoxidase activity

At the end of reperfusion, the ischaemic/reperfused cardiac tissues and serum were frozen and stored at −80°C. Myeloperoxidase (MPO) activity, an enzyme occurring almost exclusively in neutrophils, was determined by a commercial assay kit (Nanjing Jiancheng Bio-engineering Institute, China). In brief, cardiac tissue was homogenized in 50 mmol/l potassium phosphate buffer, pH 6, containing 0.5% hexadecyltrimethyl ammonium bromide. The homogenates were centrifuged for 10 min at 12,500×g at 4°C. The supernatants were collected and reacted with 0.167 g/L *o*-dianisodine dihydrochloride and 0.0005% H_2_O_2_ in 50 mmol/L phosphate buffer, and absorbance determined spectrophotometrically at 460 nm.

### Determination of NO Production

Total NO production in left ventricle of cardiac tissue was determined by measuring the concentration of nitrite, a stable metabolite of NO, with a modified Griess reaction as described [23]. The levels of NO in plasma was determined using a NO-specific microelectrode (World Precise Instrument, USA ).

### Real-time quantitative PCR

Total RNA was extracted from cardiac tissues using Trizol (Invitrogen, Carlsbad, CA) and reverse transcribed into cDNA using the Prime Script RT reagent kit (Takara Biotechnology, China) according to the manufacturer's instructions. mRNA levels of target genes were quantified using SYBR Green Master Mix (Takara Biotechnology, China) with ABI PRISM 7500 Sequence Detector system (Applied Biosystems, CA). The primer sequences were as follows: HIF-1α (forward, 5′-ACTGATTGCATCTCCACCTTCT-3′; reverse, 5′-TCGCTTCCTCTGAGCATTCT-3′); iNOS (forward, 5′-GCTACACTTCCAACGCAACA-3′; reverse, 5′-ACAATCCACAACTCGCTCCA3′); VEGF (forward, 5′-TGCACCCACGACAGAAGGG-3′; reverse, 5′-TCACCGCCTTGGCTTGTCA–CAT-3′). Samples were normalized against 18 s (forward, 5′-GTAACCCGTTGAACCCCATT-3′; reverse, 5′-CCATCCAATCGGTAGTAGCG-3′) expression to ensure equal loading.

### Western blotting

At the end of reperfusion, the hearts were removed and the ischaemic area of cardiac tissue was homogenized in lysis buffer (final composition in mmol/l: NaCl 150; TRIS 25; Na fluoride 50; Na orthovanadate 1.0; phenylmethylsulfonyl fluoride 1.0; aprotinin 1 mg/l; leupeptin 10 mg/l; pH 7.6). The homogenates were placed on ice for 40 min, then centrifuged for 10 min at 12,500× g at 4°C. The supernatants, comprising cardiac lysates, were subjected to SDS-PAGE using a 7.5% or 10% acrylamide gel, followed by Western blotting. Proteins on the gel were transferred to a polyvinylidene fluoride membrane for 2 h at 15 V. The membranes were blocked for 2 h at room temperature in TBS-Tween 20 (0.02%) containing 3% bovine serum albumin, followed by probing with anti-p85α subunit of PI3K (rabbit polyclonal, Santa Cruz Biotechnology Inc, USA), anti-phospho-eNOS (Ser^1177^) (rabbit polyclonal, Cell Signaling Technology Inc. MA, USA), anti-eNOS (mouse monoclonal, Sigma, MO, USA), anti-phospho-Akt (Ser^473^) (rabbit polyclonal, Sigma, USA), anti-Akt (rabbit polyclonal, Cell Signaling Technology Inc. MA, USA), anti-iNOS (rabbit polyclonal, Bioworld Technology Inc. MD, USA), anti-HIF-1α (rabbit polyclonal, Santa Cruz Biotechnology Inc, USA) or anti-HIF-1β (rabbit monoclonal, Cell Signaling Technology Inc. MA, USA), dilution 1∶1000 in blocking buffer at 4°C overnight. After 3 washes with TBS-Tween 20 (0.02%), the appropriate secondary antibody was added at room temperature for 2 h. Excess antibody was removed by washing 3 times with TBS-Tween 20 (0.02%). The membranes were covered with Enhanced Chemiluminescence (ECL) Western blotting detection reagents (Amersham Biosciences, NJ, USA) for 1 min, and then exposed to Hyperfilm for up to 15 min. The films were scanned into Microsoft Windows XP Paint software using a ScanJet 3400C scanner (Hewlett Packard, CA, USA). The area-density product of each band was measured using Image J 1.25 s software (National Institutes of Health, MD, USA). p-Akt or p-eNOS immunoblots were then stripped with stripping buffer (100 mM 2-mercaptoethanol; 2% (w/v) SDS; 62.5 mM Tris-HCl; pH 6.7) at 50°C for 30 min, and re-probed for total Akt or eNOS.

### Measurement of superoxide anion generation

Cardiac tissue samples were homogenized and centrifuged as described above for western blotting. The supernatant was used for measurement of superoxide anion production by lucigenin-enhanced chemiluminescence. The light reaction between superoxide and lucigenin (5 µmol/L) was detected in a 96-well microplate luminometer (GloMax, Promega, USA) during incubation in a HEPES-modified Krebs buffer (pH 7.4). Additionally, hearts removed from rats were immediately frozen in Tissue-Tek OCT embedding medium (Sakura Finetek, Tokyo, Japan), then cut into 5 µm-thick sections and placed on glass slides. Dihydroethidium (DHE, 2 µmol/L) was applied to each tissue section and the slides incubated in a light-protected humidified chamber at 37°C for 15 min. The slides were then examined by fluorescence microscopy (Olympus, Japan).

### Determination of myocardial peroxynitrite (ONOO^−^) production

Cardiac tissue samples were homogenized on ice in 20 mM HEPES buffer containing 1 mM EDTA, protease inhibitors (Roche, Germany), and PMSF 1 mM (Sigma, USA). After centrifugation (10 min, 800 g, 4°C) the supernatant was used for measurement of peroxynitrite production by luminol-enhanced chemiluminescence [24]. The light reaction between ONOO^−^ and luminol(250 µmol/L) was detected in a 96-well microplate luminometer (Biotek, USA) during incubation in a HEPES-modified Krebs buffer (pH 7.4).

### Determination of myocardial TNF-α production

Myocardial expression of TNF-α was determined using commercially available enzyme-linked immunosorbent assay kits (Bender, BMS622, Minneapolis, USA). Protein concentration in the samples was measured by BCA Protein Assay (PIERCE, USA), with bovine serum albumin used as the standard.

### Myocardial apoptosis

For terminal deoxynucleotidyl-transferase mediated dUTP nick-end labeling (TUNEL) staining, hearts were fixed in 4% v/v paraformaldehyde, embedded in paraffin, cut into 5-µm thickness sections and treated as indicated in the In Situ Cell Death Detection kit, POD (Roche, Germany). Following this, sections were co-stained with hematoxylin (Beyotime, China) to identify cardiomyocytes.

### Statistical analysis

All data are expressed as mean ± SEM. Comparisons between treatment groups were by Student's *t* test (paired or unpaired) or ANOVA, with or without repeated measures, as appropriate. Differences were considered significant at *P*<0.05 (two-tailed).

## Results

### *l*-THP dose-dependently decreases myocardial infarct size in rats following myocardial I/R

**Figure 2:**
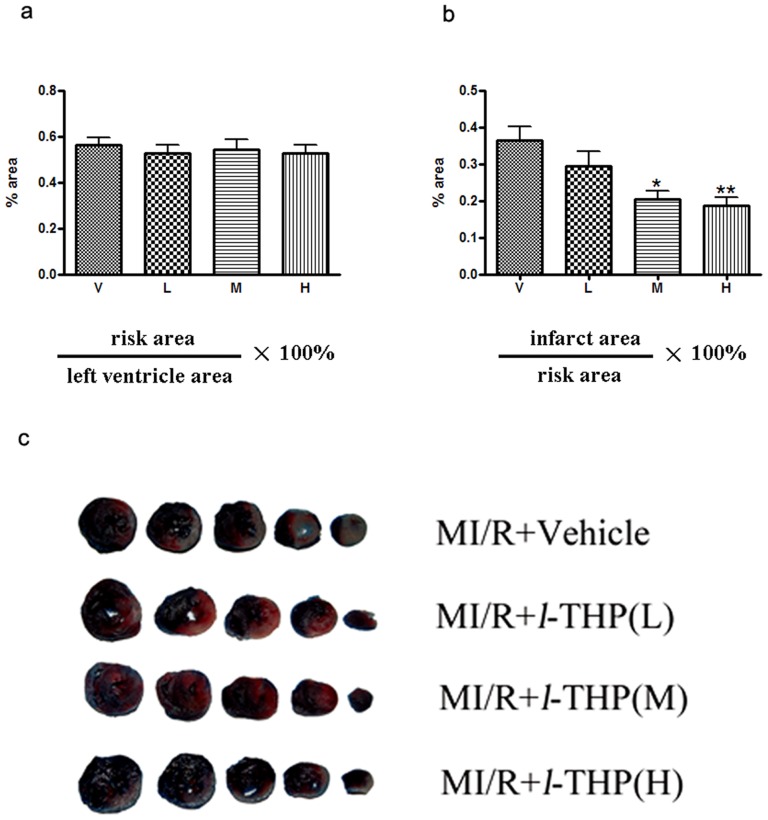
*l*-THP decreased myocardial infarct size in myocardial I/R (MI/R) rats. (**a**) RA (risk area)/LV (left ventricle area) ratio. RA/LV ratio was not changed. (**b**) IA (infarct area)/RA ratio. Compared with the vehicle group (V), IA/RA ratio was significantly lower in rats that treat with *l*-THP(M) and *l*-THP(H), *P<0.05, **P<0.01 vs. vehicle group, n = 6, but *l*-THP(L) shows no effect. (**c**) Representative heart sections, compared with vehicle, infarct area was reduced in the heart by *l*-THP (M) and *l*-THP (H).

30 min ischaemia followed by 6 h reperfusion resulted in myocardial injury, as expected (Figure 2). After I/R injury, myocardial IA/RA ratio was 36.3±3.9% in the vehicle group but was reduced to 20.5±2.2% and 18.6±2.2% after treated with *l*-THP at either a moderate dose (20 mg/kg.) or a high dose (40 mg/kg b.w.) respectively. No effect of *l*-THP on infarct size was detected at a low dose (10 mg/kg ).

### *l*-THP dose-dependently improves left ventricular function in rats following myocardial I/R

**Figure 3:**
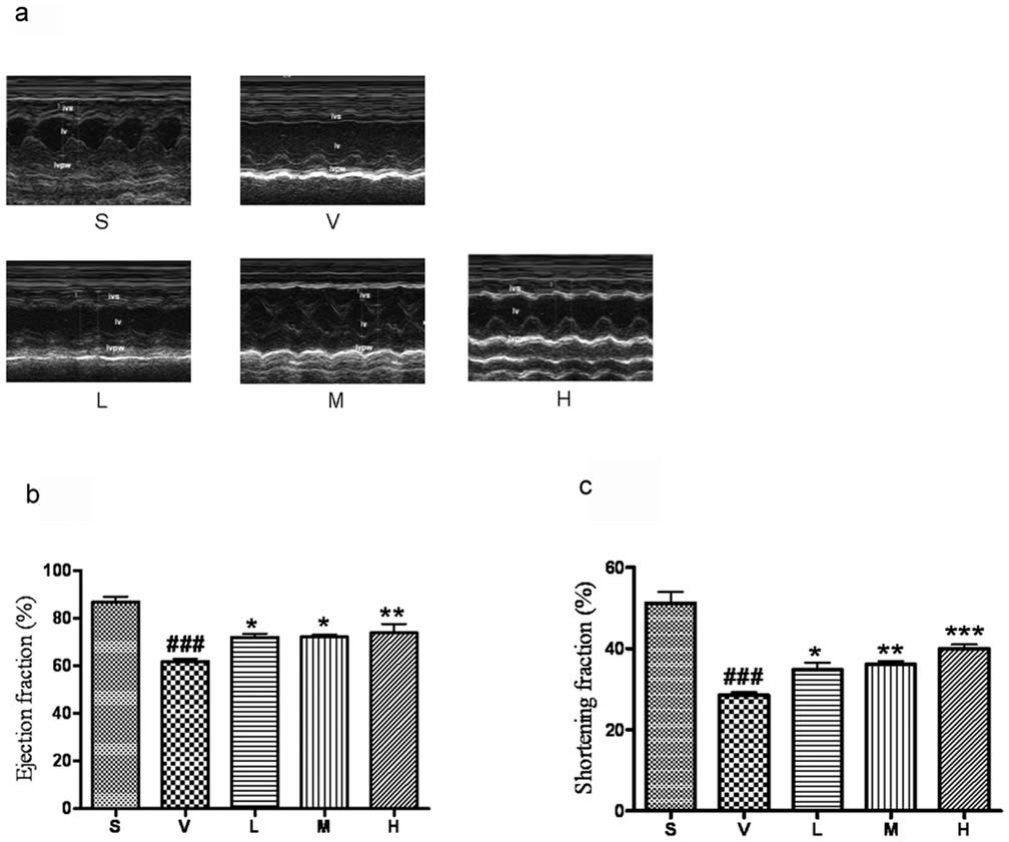
*l*-THP improves the left ventricular function of myocardial I/R rats. (**a**) Typical M-mode echocardiograms (**b, c**) I/R induced a pronounced reduction in left ventricular ejection fraction (EF) and fractional shortening (FS), both effects being prevented by *l*-THP. ^###^P<0.001 vs. sham group (S),*P<0.05,** P<0.01, ***P<0.001vs. vehicle group, n = 6.

Compared to the sham-operated group, 30 min ischaemia followed by 6 h reperfusion, ejection fraction (EF) and fractional shortening (FS) in the vehicle-treated group were significantly decreased. Treatment with *l*-THP (10, 20 and 40 mg/kg b.w.), both EF and FS following ischaemia-reperfusion were significantly increased (Figure 3).

### *l*-THP dose-dependently decreases plasma creatine kinase activity

**Figure 4:**
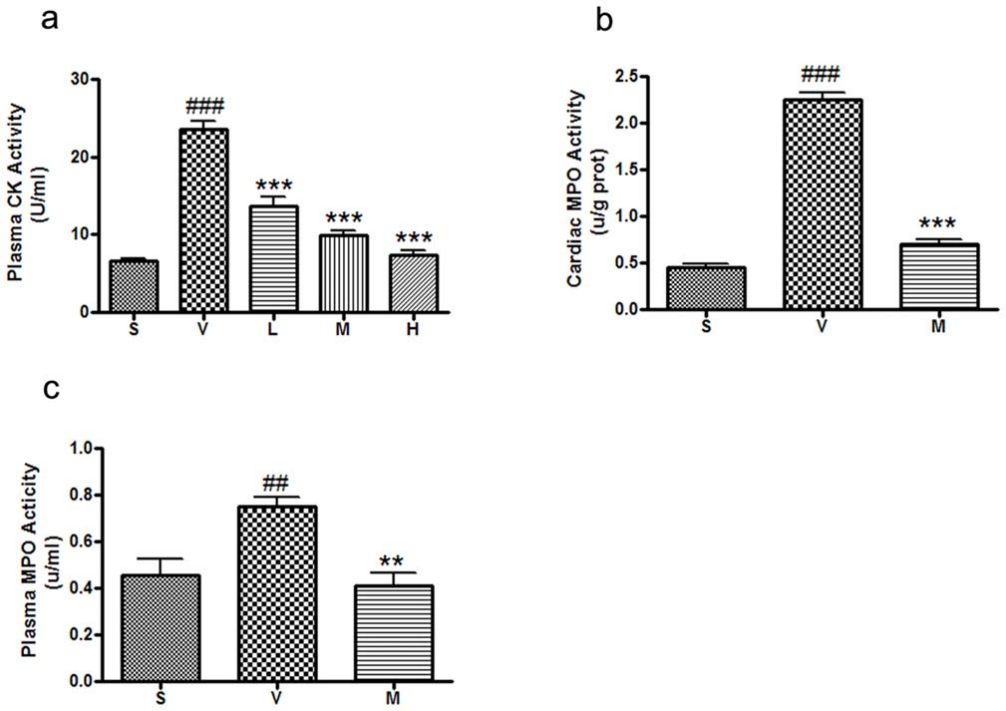
*l*-THP decreased the activity of creatine kinase and myeloperoxidase. (**a**) Plasma CK activity of myocardial I/R rats treated with vehicle and *l*-THP. Plasma CK activity was significantly decreased in rats that treat with *l*-THP, ^###^P<0.001 vs. sham group, ***P<0.001 vs. vehicle group, n = 6. (**b**) Cardiac MPO activity of myocardial I/R rats treated with vehicle and *l*-THP (M). (**c**) Plasma MPO activity of myocardial I/R rats treated with vehicle and *l*-THP (M). Compared with the vehicle group, both cardiac and plasma MPO activity was significantly decreased, ^##^P<0.01, ^###^P<0.001 vs. sham group, **P<0.01, ***P<0.001 vs. vehicle group, n = 6.

30 min ischaemia followed by 6 h reperfusion resulted in increased plasma CK activity (Figure 4a). Treatment with *l*-THP (10–40 mg/kg) progressively decreased CK activity following ischaemia-reperfusion.

### *l*-THP decreased myocardial and plasma myeloperoxidase activity

It is well established that myocardial I/R injury involves an inflammatory response where neutrophils play a critical role. 30 min of ischaemia followed by 6 h reperfusion increased MPO activity in both plasma and myocardium. *l*-THP (20 mg/kg b.w.) treatment significantly attenuated the elevation in both plasma and myocardial MPO activity, consistent with an inhibitory effect on neutrophil function (Figure 4b–c).

### *l*-THP dose-dependently decreases myocardial and plasma NO biosynthesis

**Figure 5:**
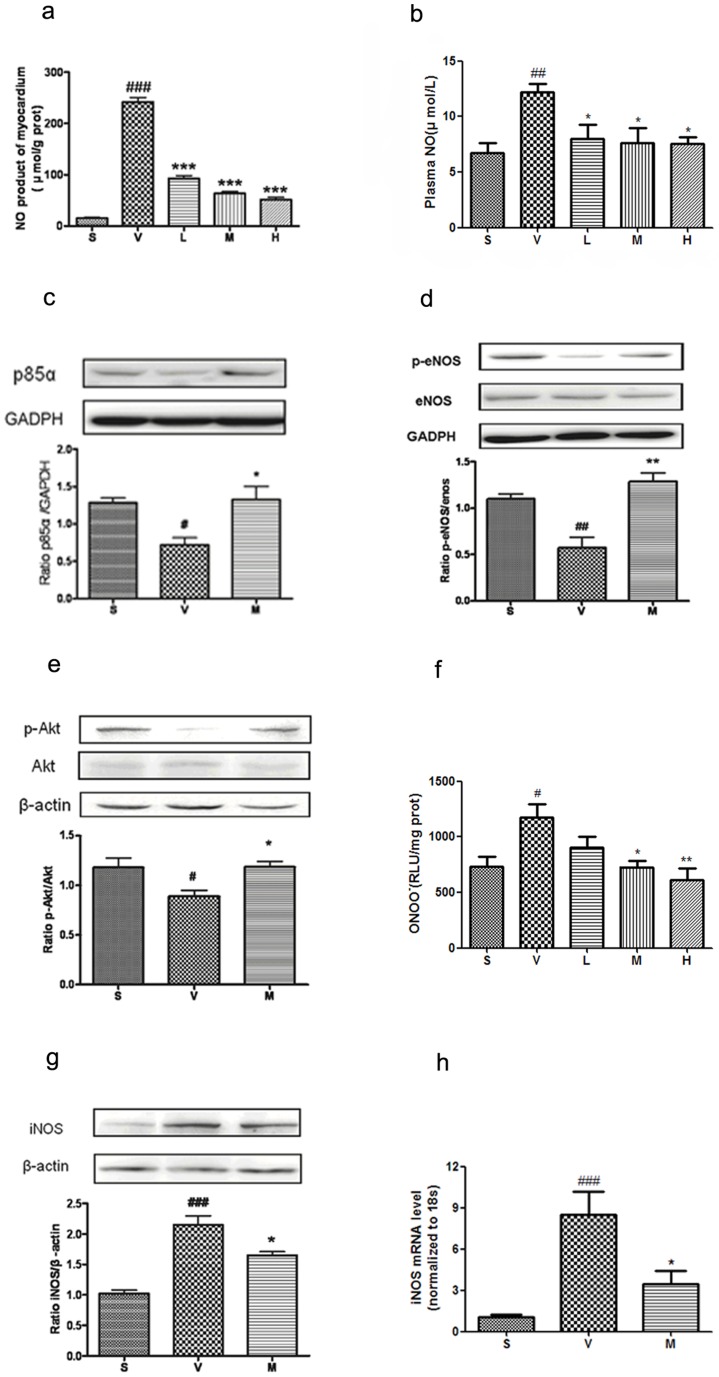
*l*-THP decreased level of NO, ONOO^−^ and iNOS, regulated expression of p85, eNOS and Akt. (**a**) Cardiac NO production concentration of myocardial I/R rats treated with vehicle, and three doses of *l*-THP. Compared with the vehicle group, NO production concentration was significantly decreased, ^###^P<0.001 vs. sham group, ***P<0.001 vs. vehicle group, n = 6. (**b**) The production of NO in plasma in myocardial I/R rats treated with vehicle, and three doses of *l*-THP. NO concentration was significantly decreased, which compared with the vehicle group, ^##^P<0.01 vs. sham group, *P<0.05 vs. vehicle group, n = 6. (**c**) p85 of myocardial I/R rats treated with vehicle and *l*-THP (M). p85 was significantly increased in *l*-THP (M)-treated group, ^#^P<0.05 vs. sham group, *P<0.05 vs. vehicle group, n = 5. (**d**) Serine^1177^ phosphorylation of eNOS of myocardial I/R rats treated with vehicle and *l*-THP (M). Serine^1177^ phosphorylation of eNOS was significantly increased in *l*-THP (M)-treated group, but total eNOS expression was not increased, ^##^P<0.05 vs. sham group, **P<0.01 vs. vehicle group, n = 5. (**e**) Serine^473^ phosphorylation of Akt of myocardial I/R rats treated with vehicle and *l*-THP (M). Serine^473^ phosphorylation of Akt was significantly increased in *l*-THP (M)-treated group, but total Akt expression was not increased, ^#^P<0.05 vs. sham group, *P<0.05 vs. vehicle group, n = 5. (**f**) Cardiac ONOO^−^ generation, as measured by luminol-enhanced chemiluminescence, in each of the five groups, ^#^P<0.05 vs. sham group, *P<0.05, **P<0.01 vs. vehicle group, n = 6. (**g**) iNOS expression of myocardial I/R rats treated with vehicle and *l*-THP (M). iNOS expression was significantly decreased in *l*-THP (M)-treated group, ^###^P<0.001 vs. sham group, *P<0.05 vs. vehicle group, n = 5. (**h**) The mRNA levels of iNOS in myocardial I/R rats treated with vehicle and *l*-THP (M). iNOS expression was significantly decreased in *l*-THP (M)-treated group, ^###^P<0.001 vs. sham group, *P<0.05 vs. vehicle group, n = 6.

NO production in myocardium and plasma of rats subjected to I/R was higher than the sham group (Figure 5a–b). Treatment with *l*-THP (10–40 mg/kg b.w.) significantly decreased myocardial NO generation following ischaemia-reperfusion.

### *l*-THP increases p85, serine^1177^ phosphorylation of eNOS and serine^473^ phosphorylation of Akt in myocardium

The PI3K-Akt-eNOS pathway has been reported to play a protective role in myocardial I/R [21]. As shown in Figure 5c, *l*-THP (20 mg/kg b.w.) significantly increased the expression of p85 in myocardium. Serine^1177^ phosphorylation of eNOS is an important post translational modification by which eNOS activity is increased in response to a variety of physiological stimuli [25]. To determine whether the decrease in myocardial NO production in response to *l*-THP might be due to inhibition of eNOS, through a decrease in its serine^1177^ phosphorylation, we examined this as well as total expression of eNOS by Western blotting of myocardial lysates. As shown in Figure 5d, *l*-THP (20 mg/kg b.w.) significantly increased eNOS serine^1177^ phosphorylation, with no corresponding alteration in total eNOS expression in myocardium. One of the most important enzymes mediating serine^1177^ phosphorylation-dependent activation of eNOS is protein kinase Akt [26]. The active form of Akt is phosphorylated at serine^473^ and threonine^308^. We therefore measured serine^473^-phosphorylated Akt as an index of Akt activation, in response to *l*-THP (20 mg/kg b.w.), by Western blotting. As shown in Figure 5e, serine^473^-phosphorylated Akt in *l*-THP group was increased significantly compared with vehicle group, but there was no corresponding change in total Akt expression in the myocardium.

### *l*-THP decrease iNOS expression and ONOO^−^ production in myocardium

Various studies have demonstrated that pathological concentrations of NO produced by iNOS may result in nitrative stress and tissue injury, largely by generating the powerful nitrative molecule peroxynitrite (ONOO^−^) [27], [28]. Accordingly, we measured ONOO^−^ levels in each group by luminol-enhenced chemiluminescence. As shown in Figure 5f, increased ONOO^−^ production was detected after cardiac I/R, which was attenuated by treatment with *l*-THP (20–40 mg/kg b.w.). To investigate whether the decrease in myocardial NO production in response to *l*-THP might be explained by a decrease in iNOS expression, we examined iNOS expression in both protein and mRNA levels. As shown in Figure 5g, *l*-THP (20 mg/kg b.w.) decreased total expression of iNOS in myocardium by western blotting. *l*-THP (20 mg/kg b.w.) also reduced the iNOS mRNA level in the myocardium after I/R (Figure 5h).

### *l*-THP affects the expression of HIF-1 and VEGF

**Figure 6:**
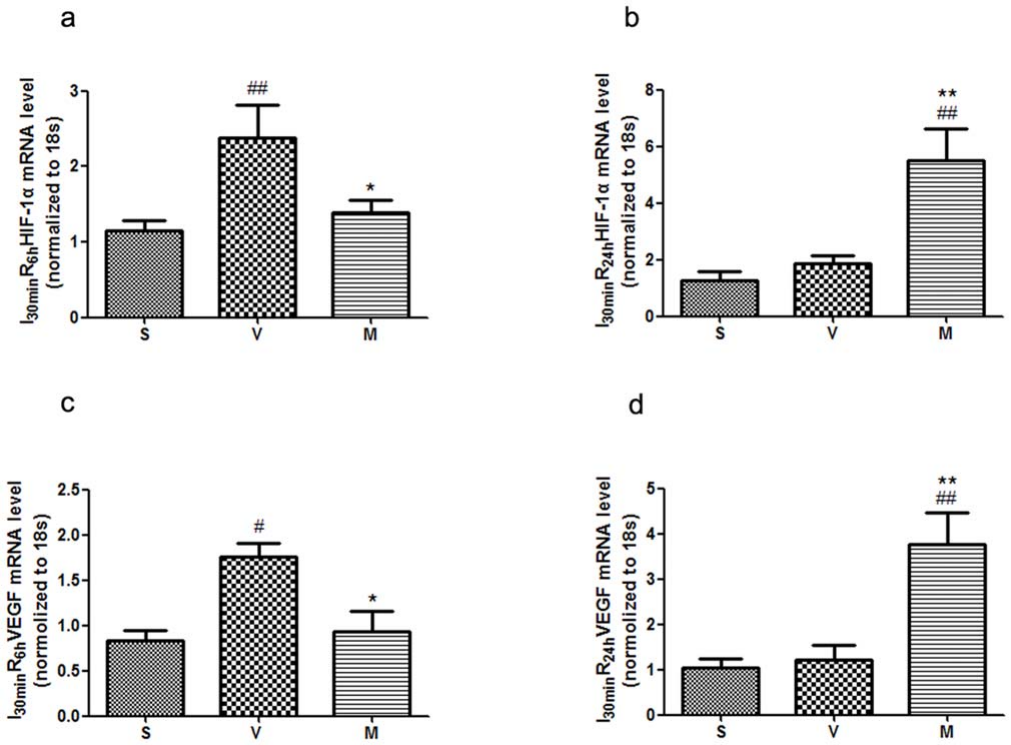
*l*-THP affected the expression of HIF-1 and VEGF. (**a**) The mRNA level of HIF-1α in I_30 min_R_6 h_. Compared with the sham group, HIF-1α expression significantly increased in vehicle group, ^##^P<0.01 vs. sham group, *P<0.05 vs. vehicle group, n = 6. (**b**) The mRNA level of HIF-1α in I_30 min_R_24 h_. Compared with the vehicle group, n = 6. (**c**) The mRNA level of VEGF in I_30 min_R_6 h_. Compared with the sham group, VEGF expression significantly increased in vehicle group, ^#^P<0.05 vs. sham group, *P<0.05 vs. vehicle group, n = 6. (**d**) The mRNA level of VEGF in I_30 min_R_24 h_. Compared with the vehicle group, VEGF expression significantly increased in *l*-THP (M)-treated group, ^##^P<0.01 vs. sham group, **P<0.01 vs. vehicle group, n = 6.

Compared with sham group, the expression of HIF-1α mRNA increased in the ischaemic region of myocardium in I_30 min_R_6 h_, but there wasn't apparent alteration in I_30 min_R_24 h_ (Figure 6a–b). Interestingly, after treatment with *l*-THP, the expression of HIF-1α mRNA increased significantly in I_30 min_R_24 h_, while it closed to the level of sham group in I_30 min_R_6 h_. The changes of VEGF mRNA in both reperfusion at 6 h and at 24 h were similar to those of HIF-1α (Figure 6c–d). This indicated that at temporary ischaemia, expression of HIF-1α and VEGF were increased, but that after a longer time of reperfusion, HIF-1α and VEGF were reduced to basal levels. *l*-THP increased the expression of HIF-1α and VEGF mRNA levels in I_30 min_R_24 h_.

### *l*-THP decreases myocardial superoxide anion

**Figure 7:**
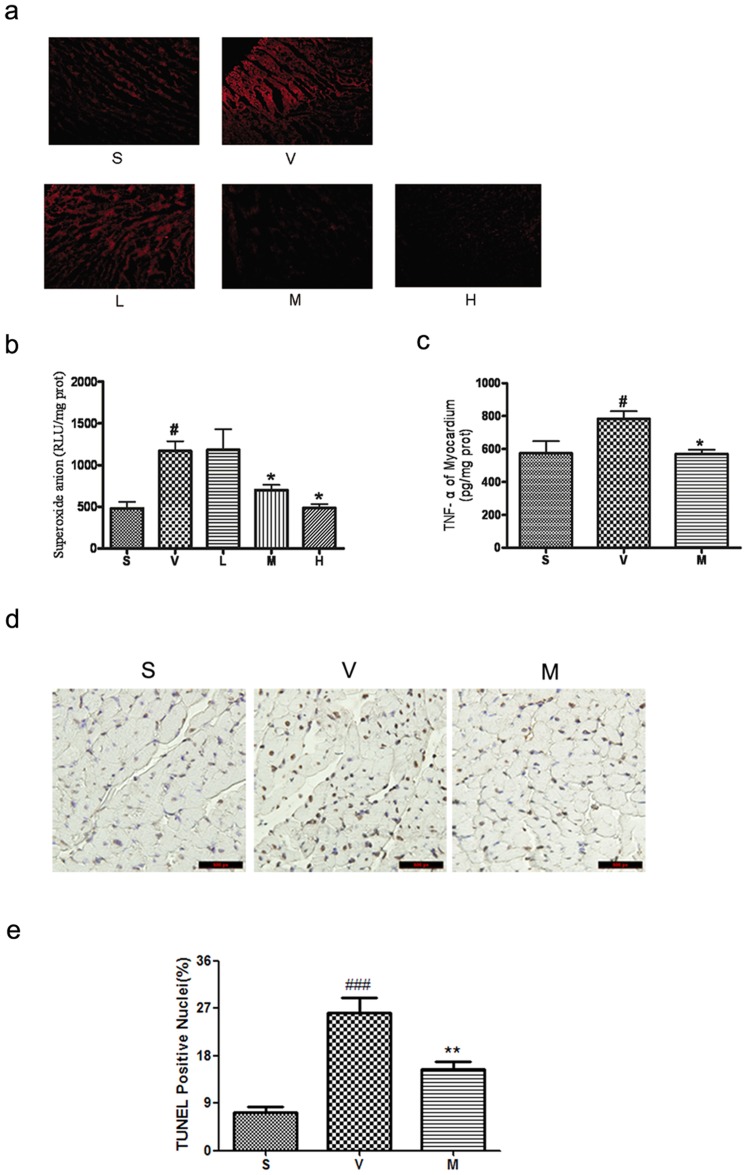
*l*-THP decreased cardiac oxidative stress, TNF-α production and myocardial apoptosis. (**a**) Representative photomicrographs of DHE-stained cardiac sections in each of the five groups. (**b**) Cardiac superoxide anion generation measured by lucigenin-enhanced chemiluminescence. Compared with the sham group, superoxide anion production significantly increased in vehicle group, while decreased in *l*-THP -treated group. ^#^P<0.05 vs. sham group, *P<0.05 vs. vehicle group, n = 6. (**c**) Cardiac TNF-α concentration of myocardial I/R rats treated with vehicle and *l*-THP (M). Compared with the vehicle group, cardiac TNF-α concentration was significantly decreased, ^#^P<0.05 vs. sham group, *P<0.05 vs. vehicle group, n = 6. (**d**) The apoptosis positive cells in myocardial of myocardial I/R rats, as determined by TUNEL assay. (**e**) Compared with the vehicle group, apoptosis positive cells was significantly increased, ^###^P<0.001 vs. sham group, **P<0.01 vs. vehicle group, n = 5.

Reactive oxygen species are believed to play a critical role in I/R injury. Fluorescence microscopy of DHE-stained cardiac sections, as well as lucigenin chemiluminescence of cardiac homogenates, revealed increased superoxide anion production after cardiac I/R, which was abolished by treatment with *l*-THP (20–40 mg/kg b.w.) (Figure 7a–b).

### *l*-THP decreases TNF-α production by myocardium

TNF-α is a multifunctional pro-inflammatory cytokine that regulates neutrophil infiltration [29]. We found that TNF-α concentration was increased in myocardium of rats subjected to myocardial I/R (Figure 7c). Treatment with *l*-THP (20 mg/kg b.w.) significantly reduced cardiac TNF-α concentration.

### *l*-THP reduced myocardial apoptosis

Because apoptosis is a major event of I/R injury, we additionally evaluated the effect of *l*-THP on apoptosis in this study. As demonstrated in Figure 7d–e, the number of TUNEL-positive cells in the ischaemic border zone was smaller in *l*-THP treated animals than vehicle-treated animals, which decreasing from 26.2±2.8% to 15.4±1.6%.

## Discussion

Myocardial infarction, and consequent loss of functional myocardium, is a major cause of heart failure. Despite interventional treatment or thrombolysis, prognosis remains poor in patients with large infarct area and/or severe left ventricular dysfunction. As well as the damage caused by ischaemia, a further volume of functional myocardium is lost immediately after reperfusion (within several hours), and this reperfusion damage is a major determinant of post-myocardial infarction. Cardioprotection before reperfusion may confer some benefit in reducing myocardial I/R injury [30], and certain drugs such as statins [31] and angiotensin receptor blockers [32], have been shown to decrease cardiovascular morbidity and mortality when administered before elective cardiac surgery or percutaneous coronary intervention.

The results of the present study indicate that the infarct size of *l*-THP-treated rats was significantly reduced compared with untreated rats whilst cardiac function was significantly improved, following myocardial ischaemia and subsequent reperfusion. Moreover, this effect was explained, in large part, by a decrease in myocardial NO production. NO plays a crucial role in many aspects of the pathophysiology of heart failure. NO has often been described as a ‘double-edged’ sword; NO inhibits I/R injury, represses inflammation, and prevents left ventricular (LV) remodeling, whereas excess NO and coexistence of reactive oxygen species (ROS) with NO are injurious [33]. NO donors have also been reported to increase cardiomyocyte death and to switch the nature of cell death from apoptosis to necrosis, in a concentration-dependent manner [34]. The detrimental effect of excessive NO is attributable to its action on mitochondria. NO inhibits the mitochondrial respiratory chain, resulting in inhibition of ATP production, as well as increase in production of reactive oxygen species and an increase in susceptibility to cell death [35], [36]. During reperfusion, due to disturbance in the redox state of the cells, excess NO can combine with superoxide anion, resulting in formation of the reactive radical peroxynitrite (ONOO^−^), which inhibits mitochondrial respiration at multiple sites, and also causes mitochondrial permeability transition pore (MPTP) opening [37], [38]. This in turn leads to membrane lipid peroxidation and in the interruption of normal signalling pathways.

The PI3K/Akt/eNOS/NO pathway has been reported to play a protective role in myocardial I/R [21]. After agonist stimulation of PI3K, cytoslic Akt translacates to the plasma menbrance where it is activated by serine and threonine phosphorylation. This membrane-activated form of Akt has been shown to phosphorylate human eNOS specifically at serine 1177, resulting in enhanced eNOS activity and increased NO release [26], [39]. Upregulation of eNOS has been reported to protect against myocardial I/R injury through suppression of vascular cell adhesion molecule expression thereby preventing excessive leukocytic tissue infiltration [40]. However, activation of iNOS, induced by pro-inflammatory cytokines, has been associated with myocardial depression [41]. Excessive generation of NO by iNOS is detrimental to cardiovascular function, as exemplified in septic shock where burst generation of iNOS-derived NO causes hypotension, cardiodepression and vascular hyporeactivity [42]. Supplementation with L-arginine under conditions where iNOS is expressed during myocardial I/R has been shown to result in a significant surge in the production of NO and ONOO^−^, which aggravate post-ischemic myocardial apoptosis [43]. A recent study has demonstrated that myocardial I/R stimulates polymorphonuclear leukocyte accumulation, resulting in myocardial injury through an iNOS-mediated mechanism involving generation of NO and ONOO^−^, and that treatment with FeTMPyP, a peroxynitrite decomposition catalyst, reduces I/R-induced, L-arginine-enhanced nitrative stress and cardiomyocyte apoptosis [44]. These studies indicate that inhibition of iNOS activity or scavenging of peroxynitrite reduces nitrative stress and thereby attenuates tissue injury during myocardial I/R In the present study, *l*-THP treatment resulted in eNOS ser^1177^ phosphorylation, via augmentation of Akt ser^473^ phosphorylation, during myocardial I/R. However, this effect is outweighed by inhibition of iNOS expression by *l*-THP during myocardial I/R, resulting in a net decrease in NO production and hence cardioprotection. Growing evidence has indicated that HIF plays a major role in myocardial I/R injury [9]. HIF-1 is a transcription factor that is expressed following a decrease in cellular oxygen pressure [8]. VEGF is a key modulator of vasculogenesis and angiogenesis in physiological and pathological conditions. VEGF is a HIF-dependent gene, which is important in I/R because of regulating collateral vessel development [9], [11]. In this study, we found that temporary ischaemia increased expression of HIF-1α and VEGF, and after a longer time of reperfusion, HIF-1α and VEGF mRNA decreased to basal level, but *l*-THP increased the expression of HIF-1α and VEGF mRNA levels.

In murine macrophages, in the absence of activation, the production of the pro-inflammatory cytokine TNF-α is repressed both at the transcriptional and translational levels [45]. However, during myocardial I/R, TNF-α and iNOS-derived NO are produced in large quantities by macrophages [46]. Inhibition of the excessive production of TNF-α and/or iNOS-derived NO can give rise to cardioprotection in such circumstances [23], [25]. Our data supported one concept that suppression of iNOS-derived NO production may contribute, at least partially, to the suppression of TNF-α production during myocardial I/R [47].

MPO is a protein present in macrophages, which can be activated by peroxynitrite which is itself a substrate for the enzyme. In addition, nitrite formed from peroxynitrite decomposition is entrapped within the phagolysosome and can serve as an additional substrate for MPO [48]. Many studies have indicated that MPO is specific for leukocytes, especially polymorphonuclear neutrophils, and MPO activity has therefore been used in many studies as an index of neutrophil accumulation in the heart [44], [49]. In the present study, we determined the activity of MPO in both plasma and myocardium, and found that *l*-THP can reduce the activity of MPO both in plasma and in myocardium, indicative of an anti-inflammatory effect, following myocardial I/R.

Studies have showed that *l*-THP can protect global cerebral ischaemia-reperfusion injury by reducing apoptosis [16], [17]. In this study, we found that *l*-THP decreased apoptosis in myocardium, indicating that *l*-THP has an anti-apoptotic effect during myocardial I/R.

In conclusion, the results of the present study indicate that *l*-THP is cardioprotective in the context of myocardial I/R, through a decrease in iNOS-mediated NO biosynthesis and consequent decreases in TNF-α synthesis and neutrophil infiltration, and lessening the extent of apoptosis. This has important potential therapeutic consequences in the context of myocardial I/R injury.

## References

[1] Griendling KK, Alexander RW (1997). Oxidative stress and cardiovascular disease.. Circulation.

[2] McCord JM (1985). Oxygen-derived free radicals in postischemic tissue injury.. N Engl J Med.

[3] Dinerman JL, Lowenstein CJ, Snyder SH (1993). Molecular mechanisms of nitric oxide regulation. Potential relevance to cardiovascular disease.. Circ Res.

[4] Radomski MW, Palmer RM, Moncada S (1987). Endogenous nitric oxide inhibits human platelet adhesion to vascular endothelium.. Lancet.

[5] Kubes P, Suzuki M, Granger DN (1991). Nitric oxide: an endogenous modulator of leukocyte adhesion.. Proc Natl Acad Sci U S A.

[6] Ma XL, Weyrich AS, Lefer DJ, Lefer AM (1993). Diminished basal nitric oxide release after myocardial ischemia and reperfusion promotes neutrophil adherence to coronary endothelium.. Circ Res.

[7] De Caterina R, Libby P, Peng HB, Thannickal VJ, Rajavashisth TB (1995). Nitric oxide decreases cytokine-induced endothelial activation. Nitric oxide selectively reduces endothelial expression of adhesion molecules and proinflammatory cytokines.. J Clin Invest.

[8] Jurgensen JS, Rosenberger C, Wiesener MS, Warnecke C, Horstrup JH (2004). Persistent induction of HIF-1alpha and -2alpha in cardiomyocytes and stromal cells of ischemic myocardium.. FASEB J.

[9] Kido M, Du L, Sullivan CC, Li X, Deutsch R (2005). Hypoxia-inducible factor 1-alpha reduces infarction and attenuates progression of cardiac dysfunction after myocardial infarction in the mouse.. J Am Coll Cardiol.

[10] Jiang BH, Rue E, Wang GL, Roe R, Semenza GL (1996). Dimerization, DNA binding, and transactivation properties of hypoxia-inducible factor 1.. J Biol Chem.

[11] Lu MJ, Chang H, Chang CC, Wang BW, Shyu KG (2005). Temporal and spatial expression of hypoxia-inducible factor-1alpha and vascular endothelial growth factor in a rat model of myocardial ischemia with or without reperfusion.. J Formos Med Assoc.

[12] Zhu XZ (1991). Development of natural products as drugs acting on central nervous system.. Mem Inst Oswaldo Cruz.

[13] Chu H, Jin G, Friedman E, Zhen X (2008). Recent development in studies of tetrahydroprotoberberines: mechanism in antinociception and drug addiction.. Cell Mol Neurobiol.

[14] Liang J, Wang FQ, Zheng PX, Liang JS (1999). Effects of dl-tetrahydropatmatine on lipid peroxidation and neuronal changes on cerebral ischemia/reperfusion injury.. Chinese Pharmacological Bulletin.

[15] Wang T, Cao XB, Sun CM, Sun SG (2003). Experimental study of protective effect of l-tetrahydropalmatine on cerebral ischemia/reperfusion injury.. Chinese Journal of Rehabilitation.

[16] Yang G, Jiang C, Tang Y, Wang P (2000). Effects of L-tetrahydropalmatine on neuron apoptosis during acute cerebral ischemia-reperfusion of rats.. J Tongji Med Univ.

[17] Liu B, Yang G (2004). Effects of L-tetrahydropalmatine on the expressions of bcl-2 and bax in rat after acute global cerebral ischemia and reperfusion.. J Huazhong Univ Sci Technolog Med Sci.

[18] Wu L, Ling H, Li L, Jiang L, He M (2007). Beneficial effects of the extract from Corydalis yanhusuo in rats with heart failure following myocardial infarction.. J Pharm Pharmacol.

[19] Ma HD, Wang YJ, Guo T, He ZG, Chang XY (2009). Simultaneous determination of tetrahydropalmatine, protopine, and palmatine in rat plasma by LC-ESI-MS and its application to a pharmacokinetic study.. J Pharmaceut Biomed.

[20] Hong ZY, Fan GR, Le J, Chai YF, Yin XP (2006). Brain Pharmacokinetics and Tissue Distribution of Tetrahydropalmatine Enantiomers in Rats after Oral Administration of the Racemate.. Biopharm Drug Dispos.

[21] Gao F, Gao E, Yue TL, Ohlstein EH, Lopez BL (2002). Nitric oxide mediates the antiapoptotic effect of insulin in myocardial ischemia-reperfusion: the roles of PI3-kinase, Akt, and endothelial nitric oxide synthase phosphorylation.. Circulation.

[22] Li C, Kao RL, Ha T, Kelley J, Browder IW (2001). Early activation of IKKbeta during in vivo myocardial ischemia.. Am J Physiol Heart Circ Physiol.

[23] Li J, Zhang H, Wu F, Nan Y, Ma H (2008). Insulin inhibits tumor necrosis factor-alpha induction in myocardial ischemia/reperfusion: role of Akt and endothelial nitric oxide synthase phosphorylation.. Crit Care Med.

[24] Radi R, Cosgrove TP, Beckman JS, Freeman BA (1993). Peroxynitrite-induced luminol chemiluminescence.. Biochem J.

[25] Sheeba MS, Asha VV (2009). Cardiospermum halicacabum ethanol extract inhibits LPS induced COX-2, TNF-alpha and iNOS expression, which is mediated by NF-kappaB regulation, in RAW264.7 cells.. J Ethnopharmacol.

[26] Dimmeler S, Fleming I, Fisslthaler B, Hermann C, Busse R (1999). Activation of nitric oxide synthase in endothelial cells by Akt-dependent phosphorylation.. Nature.

[27] Arstall MA, Sawyer DB, Fukazawa R, Kelly RA (1999). Cytokine-mediated apoptosis in cardiac myocytes: the role of inducible nitric oxide synthase induction and peroxynitrite generation.. Circ Res.

[28] Li J, Baud O, Vartanian T, Volpe JJ, Rosenberg PA (2005). Peroxynitrite generated by inducible nitric oxide synthase and NADPH oxidase mediates microglial toxicity to oligodendrocytes.. Proc Natl Acad Sci U S A.

[29] Nian M, Lee P, Khaper N, Liu P (2004). Inflammatory cytokines and postmyocardial infarction remodeling.. Circ Res.

[30] Ye Y, Lin Y, Manickavasagam S, Perez-Polo JR, Tieu BC (2008). Pioglitazone protects the myocardium against ischemia-reperfusion injury in eNOS and iNOS knockout mice.. Am J Physiol Heart Circ Physiol.

[31] Merla R, Daher IN, Ye Y, Uretsky BF, Birnbaum Y (2007). Pretreatment with statins may reduce cardiovascular morbidity and mortality after elective surgery and percutaneous coronary intervention: clinical evidence and possible underlying mechanisms.. Am Heart J.

[32] Matsuhisa S, Otani H, Okazaki T, Yamashita K, Akita Y (2008). Angiotensin II type 1 receptor blocker preserves tolerance to ischemia-reperfusion injury in Dahl salt-sensitive rat heart.. Am J Physiol Heart Circ Physiol.

[33] Otani H (2009). The role of nitric oxide in myocardial repair and remodeling.. Antioxid Redox Signal.

[34] Uchiyama T, Otani H, Okada T, Ninomiya H, Kido M (2002). Nitric oxide induces caspase-dependent apoptosis and necrosis in neonatal rat cardiomyocytes.. J Mol Cell Cardiol.

[35] Brown GC, Borutaite V (2007). Nitric oxide and mitochondrial respiration in the heart.. Cardiovasc Res.

[36] Davidson SM, Duchen MR (2006). Effects of NO on mitochondrial function in cardiomyocytes: Pathophysiological relevance.. Cardiovasc Res.

[37] Borutaite V, Morkuniene R, Brown GC (1999). Release of cytochrome c from heart mitochondria is induced by high Ca2+ and peroxynitrite and is responsible for Ca(2+)-induced inhibition of substrate oxidation.. Biochim Biophys Acta.

[38] Shiva S, Oh JY, Landar AL, Ulasova E, Venkatraman A (2005). Nitroxia: the pathological consequence of dysfunction in the nitric oxide-cytochrome c oxidase signaling pathway.. Free Radic Biol Med.

[39] Fulton D, Gratton JP, McCabe TJ, Fontana J, Fujio Y (1999). Regulation of endothelium-derived nitric oxide production by the protein kinase Akt.. Nature.

[40] Kaminski A, Pohl CB, Sponholz C, Ma N, Stamm C (2004). Up-regulation of endothelial nitric oxide synthase inhibits pulmonary leukocyte migration following lung ischemia-reperfusion in mice.. Am J Pathol.

[41] Bjorkman JA, Sutherland I, Gustafsson D, Sjoquist PO, Abrahamsson T (1991). Superoxide dismutase and catalase do not improve recovery of regional myocardial contractile function when given at the time of reperfusion after reversible regional ischemia in anesthetized dogs.. Basic Res Cardiol.

[42] Titheradge MA (1999). Nitric oxide in septic shock.. Biochim Biophys Acta.

[43] Liang F, Gao E, Tao L, Liu H, Qu Y (2004). Critical timing of L-arginine treatment in post-ischemic myocardial apoptosis-role of NOS isoforms.. Cardiovasc Res.

[44] Wang XL, Liu HR, Tao L, Liang F, Yan L (2007). Role of iNOS-derived reactive nitrogen species and resultant nitrative stress in leukocytes-induced cardiomyocyte apoptosis after myocardial ischemia/reperfusion.. Apoptosis.

[45] Park E, Schuller-Levis G, Quinn MR (1995). Taurine chloramine inhibits production of nitric oxide and TNF-alpha in activated RAW 264.7 cells by mechanisms that involve transcriptional and translational events.. J Immunol.

[46] Freeman BD, Natanson C (2000). Anti-inflammatory therapies in sepsis and septic shock.. Expert Opin Investig Drugs.

[47] Zingarelli B, Hake PW, Yang Z, O'Connor M, Denenberg A (2002). Absence of inducible nitric oxide synthase modulates early reperfusion-induced NF-kappaB and AP-1 activation and enhances myocardial damage.. FASEB J.

[48] Pacher P, Beckman JS, Liaudet L (2007). Nitric oxide and peroxynitrite in health and disease.. Physiol Rev.

[49] Gao F, Yue TL, Shi DW, Christopher TA, Lopez BL (2002). p38 MAPK inhibition reduces myocardial reperfusion injury via inhibition of endothelial adhesion molecule expression and blockade of PMN accumulation.. Cardiovasc Res.

